# A Novel Phthalimide Derivative, TC11, Has Preclinical Effects on High-Risk Myeloma Cells and Osteoclasts

**DOI:** 10.1371/journal.pone.0116135

**Published:** 2015-01-24

**Authors:** Maiko Matsushita, Yoshie Ozaki, Yuka Hasegawa, Fukiko Terada, Noriko Tabata, Hirokazu Shiheido, Hiroshi Yanagawa, Tsukasa Oikawa, Koichi Matsuo, Wenlin Du, Taketo Yamada, Masashi Hozumi, Daiju Ichikawa, Yutaka Hattori

**Affiliations:** 1 Clinical Physiology and Therapeutics, Faculty of Pharmacy, Keio University, Tokyo, Japan; 2 Department of Biosciences and Informatics, Faculty of Science and Technology, Keio University, Yokohama, Japan; 3 Cell and Tissue Biology, School of Medicine, Keio University, Tokyo, Japan; 4 Department of Pathology, School of Medicine, Keio University, Tokyo, Japan; Faculté de médecine de Nantes, FRANCE

## Abstract

Despite the recent advances in the treatment of multiple myeloma (MM), MM patients with high-risk cytogenetic changes such as t(4;14) translocation or deletion of chromosome 17 still have extremely poor prognoses. With the goal of helping these high-risk MM patients, we previously developed a novel phthalimide derivative, TC11. Here we report the further characterization of TC11 including anti-myeloma effects *in vitro* and *in vivo*, a pharmacokinetic study in mice, and anti-osteoclastogenic activity. Intraperitoneal injections of TC11 significantly delayed the growth of subcutaneous tumors in human myeloma-bearing SCID mice. Immunohistochemical analyses showed that TC11 induced apoptosis of MM cells *in vivo*. In the pharmacokinetic analyses, the C_max_ was 2.1 μM at 1 h after the injection of TC11, with 1.2 h as the half-life. TC11 significantly inhibited the differentiation and function of tartrate-resistant acid phosphatase (TRAP)-positive multinucleated osteoclasts in mouse osteoclast cultures using M-CSF and RANKL. We also revealed that TC11 induced the apoptosis of myeloma cells accompanied by α-tubulin fragmentation. In addition, TC11 and lenalidomide, another phthalimide derivative, directly bound to nucleophosmin 1 (NPM1), whose role in MM is unknown. Thus, through multiple molecular interactions, TC11 is a potentially effective drug for high-risk MM patients with bone lesions. The present results suggest the possibility of the further development of novel thalidomide derivatives by drug designing.

## Introduction

Multiple myeloma (MM) is a neoplasm of plasma cells that is accompanied by various clinical manifestations including lytic bone lesions, hypercalcemia, renal dysfunction, immunodeficiency, and anemia [1, 2]. Despite recent advances in the use of newly developed drugs including immune-modulatory drugs (IMiDs) such as thalidomide, lenalidomide, and pomalidomide and proteasome inhibitors such as bortezomib, carfilzomib, and MLN9708, MM is still an incurable disease [3–7]. In particular, MM patients harboring 17p deletion, t(14;16), t(14;20), or t(4;14) are classified as a high-risk group and have shown significantly shorter survival [8–10]. For example, it is reported that even lenalidomide plus dexamethasone or bortezomib could not substantially improve the survival of refractory patients with del 17p [11, 12]. With the goal of helping prolong the survival of these high-risk MM patients, we screened 29 synthetic phthalimide derivatives and found a novel compound, 2-(2,6-diisopropylphenyl)- 5-amino-1*H*-isoindole-1,3-dione (TC11), which induced the apoptosis of KMS34 cells with t(4;14) and del17p13 [13].

Bone lytic lesions, which are seen in 80%–90% of MM patients, have clinical manifestations that include pain, pathologic fractures, spinal cord compression, and hypercalcemia, thus providing a negative impact on the quality of life of MM patients [1]. In the clinical setting, bisphosphonate, radiotherapy, and surgery are used to treat bone disease. Bisphosphonate can significantly reduce skeletal-related events in MM patients [14], but side effects such as renal impairment and osteonecrosis of the jaw are seen in some patients [15, 16]. The development of novel agents to effectively treat bone lesions without severe side effects is thus necessary.

In the present study, we evaluate the anti-myeloma effects of TC11 *in vitro* and *in vivo*, and we investigated the effects of TC11 on the differentiation of osteoclasts to determine whether this new drug could be effective for treating high-risk MM patients with bone lesions. We also examined nucleophosmin-1 (NPM1) as a molecule that binds directly to phthalimide, and the results raised the possibility that α-tubulin is involved in the anti-myeloma effect of TC11.

## Materials and Methods

### Cell lines

Human myeloma cell lines KMS11, KMS 26, KMS28, and KMS34 were established by Dr. T. Otsuki (Kawasaki Medical School, Kurashiki, Japan) from Japanese patients [17, 18] and these cell lines were kindly provided by him. MUM24 was established from a patient with thalidomide-resistant MM [19]. These cell lines were maintained in RPMI1640 medium (Sigma-Aldrich, St. Louis, MO) containing 10% fetal bovine serum (FBS). The human macrophage cell line RAW264.7 was purchased from American Type Culture Collection (Rockville, MD) and cultured in DMEM medium (Sigma-Aldrich) containing 10% FBS (Hyclone Laboratories, Logan, UT).

### Reagents

**Figure 1:**
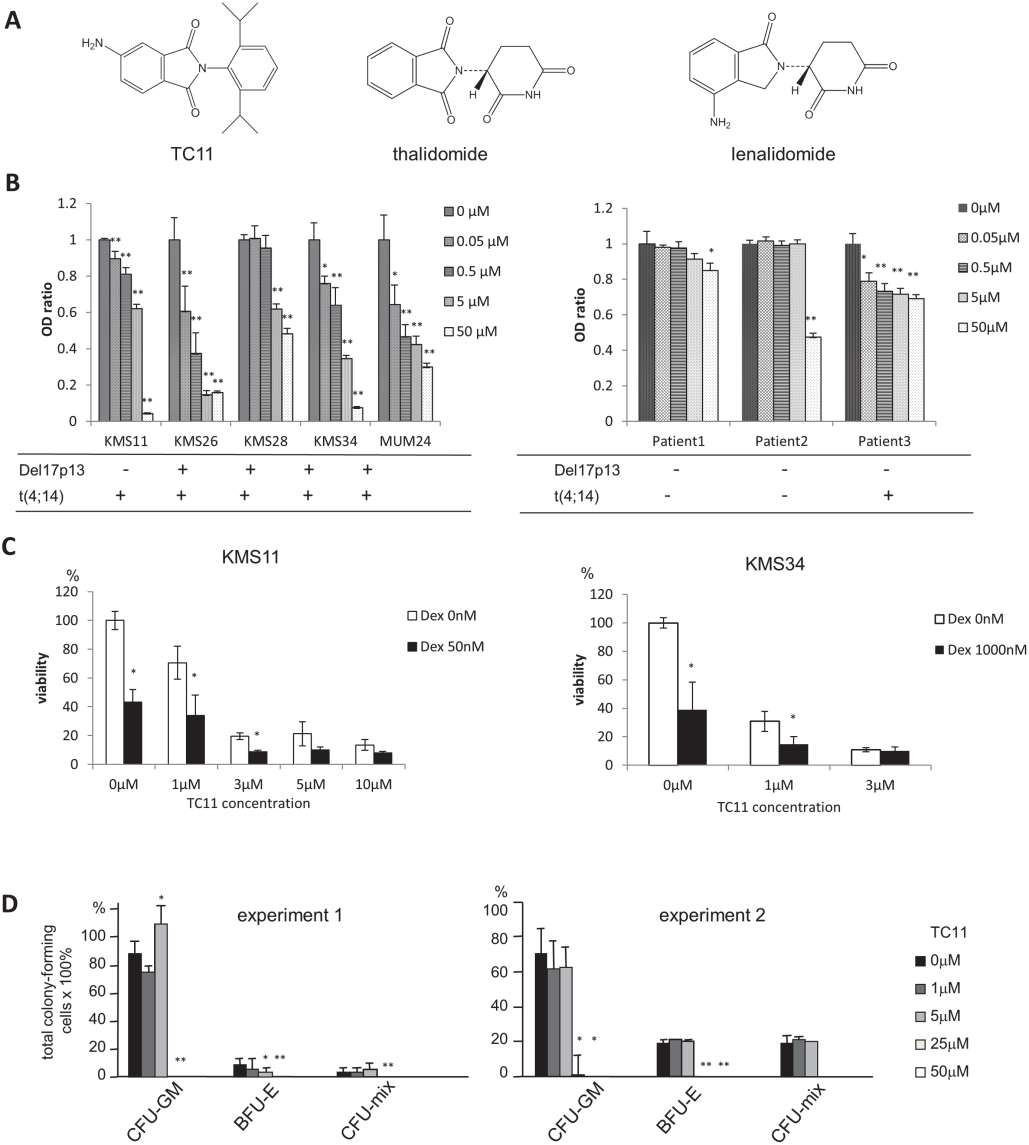
TC11 inhibited the growth of MM cell lines. (A) The chemical structures of TC11, thalidomide, and lenalidomide. (B) The dose-dependent inhibition of MM cells by TC11. Cell lines KMS11, KMS26, KMS28, KMS34, MUM24 with del 17p13 and/or t(4;14) were cultured for 48 h with the indicated dose of TC11, followed by an assessment of cell viability using MTT assays. Mononuclear cells separated from bone marrow samples of three MM patients were also treated with TC11 and examined by MTT assay. (C) Synergistic effects of TC11 with dexamethasone were examined by MTT assay. Bars indicate means±SD. *p<0.05 (Student’s *t*-test). (D) The effect of TC11 on the number of hematopoietic progenitor cells was examined by colony assay. Mice bone marrow cells were plated in 1.2% methylcellulose medium containing 20 ng/mL mIL-3, 10 ng/mL mIL-6, 20 ng/mL mSCF and 1 U/mL hEPO in the presence or absence of TC11. The numbers of colony-forming cells were counted after 14 days. Bars indicate means±SD. *p<0.05, **p<0.01 (Student’s *t*-test).

We synthesizedTC11 [2-(2,6-diisopropylphenyl)-5-amino-1*H*-isoindole-1,3-dione] from phthalic acid anhydride with a nitro group and amines, followed by catalytic hydrogenation under a nitrogen atmosphere. The benzene ring moiety of TC11 is different from those of thalidomide and lenalidomide (Fig. 1A). Dexamethasone sodium phosphate (MSD K.K., Tokyo) was used for *in vitro* proliferation assay.

### Patients’ samples

Bone marrow samples were obtained from patients with MM treated in Keio University Hospital. Mononuclear cells were isolated by Ficoll density gradient centrifugation and cryopreserved in a liquid nitrogen tank until further use. All samples contained more than 30% myeloma cells. This study was approved by the ethics committee of the Keio University School of Medicine (99–7 and 15–21) and Faculty of Pharmacy (G110107–1). Written informed consent was obtained from all patients.

### Cell viability assay

MM cells (2×10^4^ cells per well) were seeded in 96-well plates and incubated with various concentrations of TC11 (0–50 µM) at 37°C for 48 h. The number of viable cells was assessed by MTT dye absorbance (Roche Diagnostics, Indianapolis, IN) according to the manufacturer’s instructions.

### Colony-forming cell assay

To evaluate the hematological toxicity of TC11, 4×10^4^ cells/mL of bone marrow cells from 13-wk-old male ICR mice were cultured in methylcellulose medium (Stem Cell Technologies, Vancouver, BC) containing FBS, 2-mercaptoethanol, 20 ng/mL mouse stem cell factor (mSCF), 20 ng/mL mouse interleukin 3 (mIL-3), 10 ng/mL mouse interleukin-6 (mIL-6), and 1 U/mL human erythropoietin (hEPO) (kindly provided by Kyowa Hakko Kirin Co., Tokyo) in the presence or absence of TC11. On day 14, various types of colony-forming cells were counted.

### *In vivo* tumor growth assay

All of the animal experiments were approved by the Ethics Committee for Animal Experiments at Keio University Faculty of Pharmacy (Approval no.09118-(0), 09118-(1)). The *in vivo* tumor-inhibitory activity assay was performed as described with several modifications [18]. Briefly, 3×10^7^ KMS34 or KMS11 cells were subcutaneously inoculated into 5-wk-old male ICR/SCID mice (Clea Japan, Tokyo) and plasmacytoma developed in 4–7 wks. In addition, twenty mg/kg of TC11 dissolved in 10% DMSO (Sigma-Aldrich)-1% Tween80 at the concentration of 2.5 mg/mL or only 10% DMSO-1% Tween80 as a control was injected intraperitoneally twice every 3 days for 15 days (n = 7). The tumor volume was calculated according to the following formula as described [18]: width × length^2^ × 0.52.

### Histopathologic examination

The histopathologic analysis was performed as described with several modifications [18]. When the subcutaneous tumors reached 50 mm^3^, the intraperitoneal injections of TC11 was started. After 14 days of observation, the mice were sacrificed and the isolated tumors were fixed with 10% formalin and embedded in paraffin. Sliced sections were stained with hematoxylin and eosin (H. E.). Anti-human cleaved PARP (Asp214) polyclonal antibody (Cell Signaling Technology Japan, Tokyo), anti-cleaved caspase-3 (Asp175) polyclonal antibody (Cell Signaling Technology Japan) and anti-human Ki-67 monoclonal antibody (clone MIB-1) (Dako Japan, Tokyo) were used for immunohistochemistry.

### Pharmacokinetics study

To evaluate the pharmacokinetics of TC11, we obtained peripheral blood with a heparinized needle from the tail veins of 5-wk-old male ICR mice at 0.5, 1, 1.5, 4, 8, 12, and 24 h after an injection of a low dose (20 mg/kg) or a high dose (100 mg/kg) of TC11. Blood samples were centrifuged immediately at 3400*g* for 15 min at 4°C. The plasma fraction was transferred to a polypropylene tube and stored at −80°C until the assay. The plasma samples were thawed and diluted with 10% ethanol in phosphate-buffered saline (PBS). A stock solution of TC11 was prepared in ethanol at 1 mg/mL. A series of standard solutions at designated concentrations were prepared by diluting the stock solution with ethanol. All of the samples were analyzed by high-pressure liquid chromatography (HPLC; a Jasco HPLC system, Jasco, Tokyo). The C18 column (Sep-Pak; Waters Associates, Milford, MA) was used. The mobile phases were acetonitrile and 25 mM ammonium acetate (60:40).

### Osteoclast differentiation assay

We prepared murine osteoclasts from bone marrow cells as described [20]. In brief, cells obtained from the bone marrow of 5-wk-old male ICR mice were cultured in α-MEM containing 10% FBS with macrophage-colony stimulating factor (M-CSF; R&D Systems, Minneapolis, MN) (10 ng/mL). After 3 days of culture, we removed the floating cells and used the attached cells including bone marrow-derived macrophages (BMMs) as osteoclast precursors. To generate osteoclasts, BMMs were further cultured with M-CSF (10 ng/mL) and receptor activator of nuclear factor κB ligand (RANKL; R&D Systems) (10 ng/mL). After an additional 3–6 days of culture, the cells were fixed and stained for tartrate-resistant acid phosphatase (TRAP) as described [20]. TRAP-positive multinucleated cells containing more than three nuclei were considered TRAP^+^ multinuclear osteoclasts (TRAP^+^ MNCs).

### Pit formation assay

RAW 264.7 cells were incubated for 5–8 days with RANKL (10 ng/mL). After maturation into osteoclasts, the cells were seeded on BioCoat Osteologic multi-test slides (BD Falcon, BD Biosciences, San Jose, CA). Various concentrations of TC11, thalidomide (Wako, Osaka, Japan), bortezomib (Toronto Research Chemicals Inc., ON, Canada), and osteoprotegerin (OPG; R&D Systems) were added every 2 days for 7 days. Finally Von Kossa stain was conducted to visualize resorption pits. The resorption pits were observed by fluorescence microscopy (BZ-9000, Keyence, Tokyo). The pit area was quantified using Image J software (NIH).

### Immunocytochemistry

KMS34 cells were treated with 5 µM TC11 for 4 h and attached to a slide using CYTOSPIN4 (Thermo Fisher Scientific, Rockford, IL), then fixed with 2% paraformaldehyde and permeabilized with 0.5% Triton X followed by blocking with 1% bovine serum albumin (BSA) in PBS. The sample was stained with antibody against α-tubulin (Sigma-Aldrich) followed by FITC-conjugated anti-mouse IgG (Takara Bio, Shiga, Japan), and then observed by fluorescence microscopy (BZ-9000).

### Surface plasmon resonance (SPR) analysis

We determined the binding kinetics by a surface plasmon resonance (SPR) analysis with the Biacore 3000 system (GE Healthcare, Buckinghamshire, UK). All experiments were performed at 25°C using TBS buffer (25 mM Tris-HCl, pH 7.4, 137 mM NaCl, 3 mM KCl). Recombinant NPM1 was expressed in *E. coli* and purified. The NPM1 obtained (1.84 µM, 100 µL) was immobilized onto the sensor chip NTA (GE Healthcare). The measurements were performed under conditions of 662 resonance units of the ligand and at a flow rate of 30 µL/min. To determine the dissociation constants, we injected three different concentrations of TC11 and lenalidomide (Santa Cruz Biotechnology, Santa Cruz, CA). The injection periods for association and dissociation were 60 and 300 s, respectively. The binding data were analyzed with 1:1 binding with the mass transfer model in the BIA evaluation software ver. 4.1 (Biacore).

### Statistical analysis

The significance of differences was determined using Student’s *t*-test. The level of significance was specified as p<0.05.

## Results

### TC11 inhibited the growth of MM cells with chromosomal abnormalities *in vitro*

We examined whether TC11 could inhibit the growth of four different MM cell lines harboring del 17p and/or t(4;14) in addition to previously reported KMS34 cells [13]. We also analyzed TC11’s effect on the bone marrow cells obtained from three MM patients. In an MTT assay, TC11 inhibited the proliferation of the KMS11, KMS26, KMS28, and MUM24 cells as well as the proliferation of all of the bone marrow cells from the MM patients, in a dose-dependent manner (Fig. 1B). TC11 was effective both for high risk and standard risk myeloma cells. As shown in Fig. 1C, dexamethasone (Dex) potentiated TC11 anti-myeloma effect of TC11 in KMS 11 as well as KMS34 cells.

### Suppression of the growth of normal hematopoietic cells by TC11

To evaluate the effect of TC11 on normal hematopoiesis, we conducted a colony formation assay using bone marrow cells from ICR mice (Fig. 1D). 1–5 µM of TC11 (IC50 values of most MM cell lines were 3–5 µM) did not suppress the production of the total colonies of normal hematopoietic cells. 25 µM or higher concentrations of TC11 almost completely inhibited the colony formation. TC11 did not inhibit the production of colony-forming unit-granulocyte macrophage (CFU-GM), even though the number of burst-forming unit-erythroid (BFU-E) was decreased by the addition of a 5 µM of TC11 (p<0.05) (Fig. 1D).

### TC11 inhibited the growth of MM cells in the xenograft mouse model through the induction of apoptosis *in vivo*

**Figure 2:**
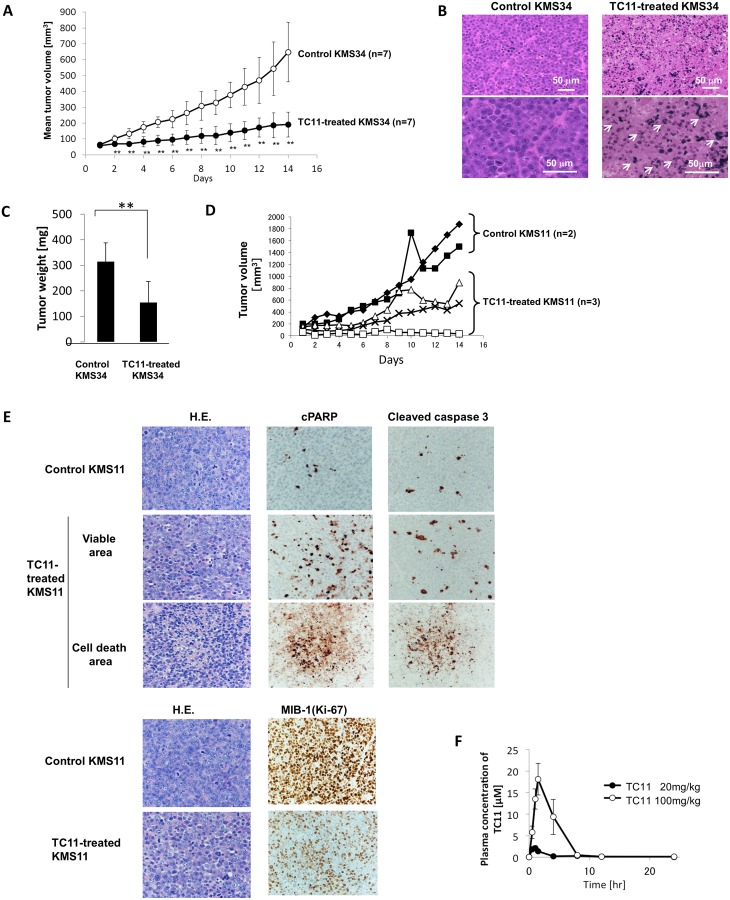
*In vivo* growth inhibition of myeloma cells by intraperitoneal injections of TC11. A total of 3×10^7^ KMS34 or KMS11 cells were inoculated subcutaneously into ICR/SCID mice. When the tumors reached 50 mm^3^ (day 0), TC11 (20 mg/kg or 100 mg/kg) or vehicle was administered intraperitoneally twice every 3 days for 2 wks. (A) Growth of KMS34-derived subcutaneous plasmacytomas was examined (n = 7). (B) Sections of dissected KMS34-derived tumors were stained with hematoxylin and eosin. The arrows indicate nuclear fragmentation of TC11-treated cells. (C) The weights of KMS34-derived tumors dissected from the KMS34-innoculated mice were measured on day 14. Bars: means ±SD.**p<0.01 (Students *t*-test). (D) Growth of KMS11-derived subcutaneous plasmacytomas was examined. (E) Pathological examination of KMS11-derived tumors was shown. Immunohistochemical staining of the KMS11-derived tumors were also shown. (F) Pharmacokinetics of TC11. Plasma concentrations of TC11 in mice after a single injection of 20 mg/kg or 100 mg/kg of TC11 determined by HPLC.

We evaluated the anti-myeloma effect of TC11 *in vivo* in the KMS34-bearing xenograft model. KMS34 tumor xenografts (~50 mm^3^) were treated with intraperitoneal injections of 20 mg/kg TC11 twice every 3 days for 2 wks, followed by a time-course analysis of the tumor volumes for 15 days. Fig. 2A shows that the tumor growth was significantly suppressed by the TC11 injections compared to the control (p<0.01). The average weight of the tumors of the TC11-treated mice on day 14 was 153.94 mg, whereas that of the control mice was 314.14 mg (p<0.01) (Fig. 2C). In addition, neither weight loss nor organ damage in lung, kidney or liver was observed macroscopically after the administration of TC11 (data not shown). Higher dose of TC11 (100 mg/kg) was injected to the xenograft model derived from another myeloma cell line, KMS11. As shown in Fig. 2D, TC11 significantly delayed the tumor growth. In one xenograft model, the tumor had almost disappeared after injection of TC11. During treatment of higher dose of TC11, significant body weight loss was not observed in TC11-treated mice.

The hematoxylin-eosin (H.E.) staining of KMS34 and KMS11-derived tumor tissues revealed elevated numbers of cells with aggregated chromatin in the TC11-treated mice compared to the untreated mice (Fig. 2B and 2E). Immunohistochemical analyses showed that cleaved PARP and cleaved caspase 3 were intensely stained in TC11-treated KMS11 tumors, especially in the cell death area (Fig. 2E). Ki-67 antigen, which is preferentially expressed during all active phases of the cell cycle but is absent in resting cells, was less weakly stained in TC11-treated tumors (Fig. 2E). These results suggested that TC11 exhibited anti-tumor activity *in vivo* through the inhibition of cell proliferation and the induction of apoptosis of myeloma cells.

### Pharmacokinetics of TC11 in the mouse model

For the pharmacokinetic study, we examined the plasma concentrations of TC11 in mice after a single injection of 20 mg/kg or 100 mg/kg of TC11 using HPLC (Fig. 2F). The maximum concentration (C_max_) was 2.1 µM, which was observed at 1 hr after the injection (T_max_). The plasma concentration of TC11 gradually decreased to 0.25 µM at 4 h and to 0.04 µM at 24 h. The elimination half-life (T1/2) was estimated to be 1.2 h. When a higher dose of TC11 (100 mg/kg) was injected, the C_max_ was 18.1 µM and the T_max_ was 1.5 h; the plasma concentration was 0.45 µM at 8 h and 0.16 µM at 24 h. The T1/2 was estimated to be 2.6 h.

### TC11 inhibited the differentiation of osteoclasts and bone resorption

**Figure 3:**
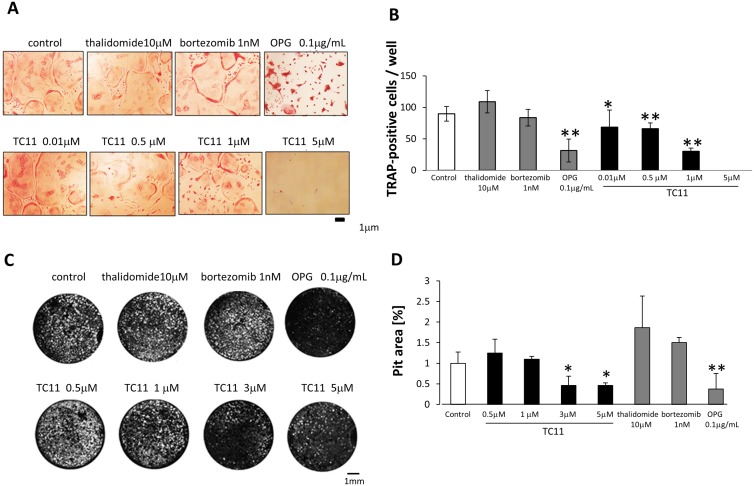
TC11 inhibited both the maturation and function of bone marrow-derived osteoclasts. Mice bone marrow cells were cultured with M-CSF (10 ng/mL) for 3 days, then further cultured with M-CSF (10 ng/mL) and RANKL (10 ng/mL). After an additional 3–6 days of culture with the indicated concentration of TC11, thalidomide, bortezomib, or DMSO, the cells were fixed and stained for TRAP as described in the Materials and Methods section. TRAP-positive multinucleated cells containing more than three nuclei were considered TRAP^+^ multinuclear osteoclasts. (A) The number and size of TRAP^+^ multinuclear osteoclasts were decreased by treatment with 0.5–1µM of TC11. (B) The number of TRAP^+^ multinuclear osteoclasts in each well of a 96-well plate was counted. Bars: means±SD. *p<0.05, **p<0.01 (Student’s *t*-test). (C) RAW 264.7 cells were incubated for 5–8 days with RANKL (10 ng/mL) and seeded on multi-test slides. The indicated concentrations of TC11, thalidomide, bortezomib, or OPG were added every 2 days for 7 days, followed by Von Kossa staining. The resorption pits were observed by fluorescence microscopy and the number of the pits was quantified using Image J software. (B) Bone resorption is indicated by white spots (pit). Bone resorption was suppressed by treatment with 3–5 µM of TC11. (D) The pit area was measured by ImageJ software. The pit area of the DMSO-treated wells was used as the control ( = 1). Data shown are representative of three independent experiments. Bars: means±SD. *p<0.05, **p<0.01 (Student’s *t*-test)

The hyperactivity of osteoclasts is considered one of the causes of lytic bone lesions, suggesting that the control of osteoclast activity could be a key to treating bone lesions in MM patients. We therefore examined the effects of TC11 on the differentiation and function of osteoclasts using mouse bone marrow primary cell culture. We analyzed M-CSF and RANKL-treated osteoclasts by TRAP staining after treatment with various concentrations of TC11 (0.01µM–5 µM) (Fig. 3A). The number of TRAP-positive multinuclear cells was significantly lower in the cells treated with more than 0.5 µM of TC11 (Fig. 3B). In contrast, 10 µM of thalidomide or 1 nM of bortezomib did not affect the number of TRAP-positive cells.

We next examined TC11’s effect on the bone resorption activity of TC11 in a pit formation assay (Fig. 3C). After the treatment of RAW264.7 cells with TC11 (3 µM, 5 µM), the bone resorption area was reduced in a dose-dependent manner (Fig. 3D). Treatment with thalidomide (10 µM) or bortezomib (1 nM) did not change the resorption area compared to the control.

### TC11 induced the fragmentation of α-tubulin

**Figure 4:**
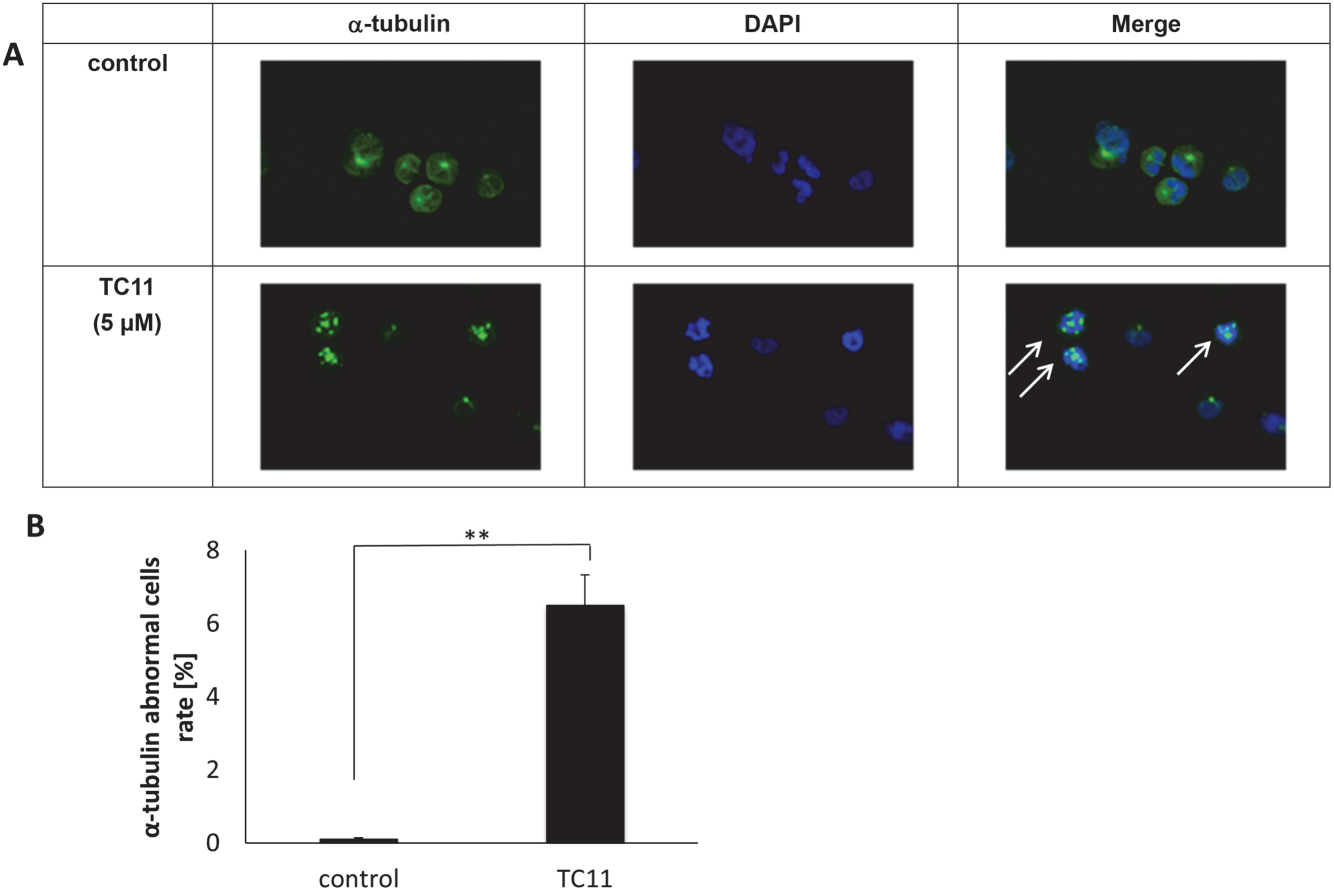
TC11 altered the α-tubulin formation of myeloma cells. KMS34 was treated with 5µM TC11 for 4 hours and attached to a slide by cytospin. The sample was fixed and stained with antibody against α-tubulin followed by FITC-conjugated anti-mouse IgG (green). Nucleus was stained with DAPI (blue). A, Representative mitotic cells with or without TC11 treatment. B, The percentage of cells with fragmented α-tubulin in 2500 cells was calculated independently in 9 areas. **P<0.01 (Student *t*-test). A and B indicate representative data from 3 experiments.

Because we previously found that TC11 binds to α-tubulin, we examined the tubulin formation in KMS34 cells after TC11 treatment. The immunohistochemical analysis showed that TC11-treated cells exhibited elevated levels of α-tubulin fragmentation (Fig. 4A). The percentage of cells with tubulin fragmentation was 6.5% in the cells treated with TC11, which is significantly higher compared to the 0.8% in the untreated cells (Fig. 4B).

### Binding between NPM and TC11 or lenalidomide

**Figure 5:**
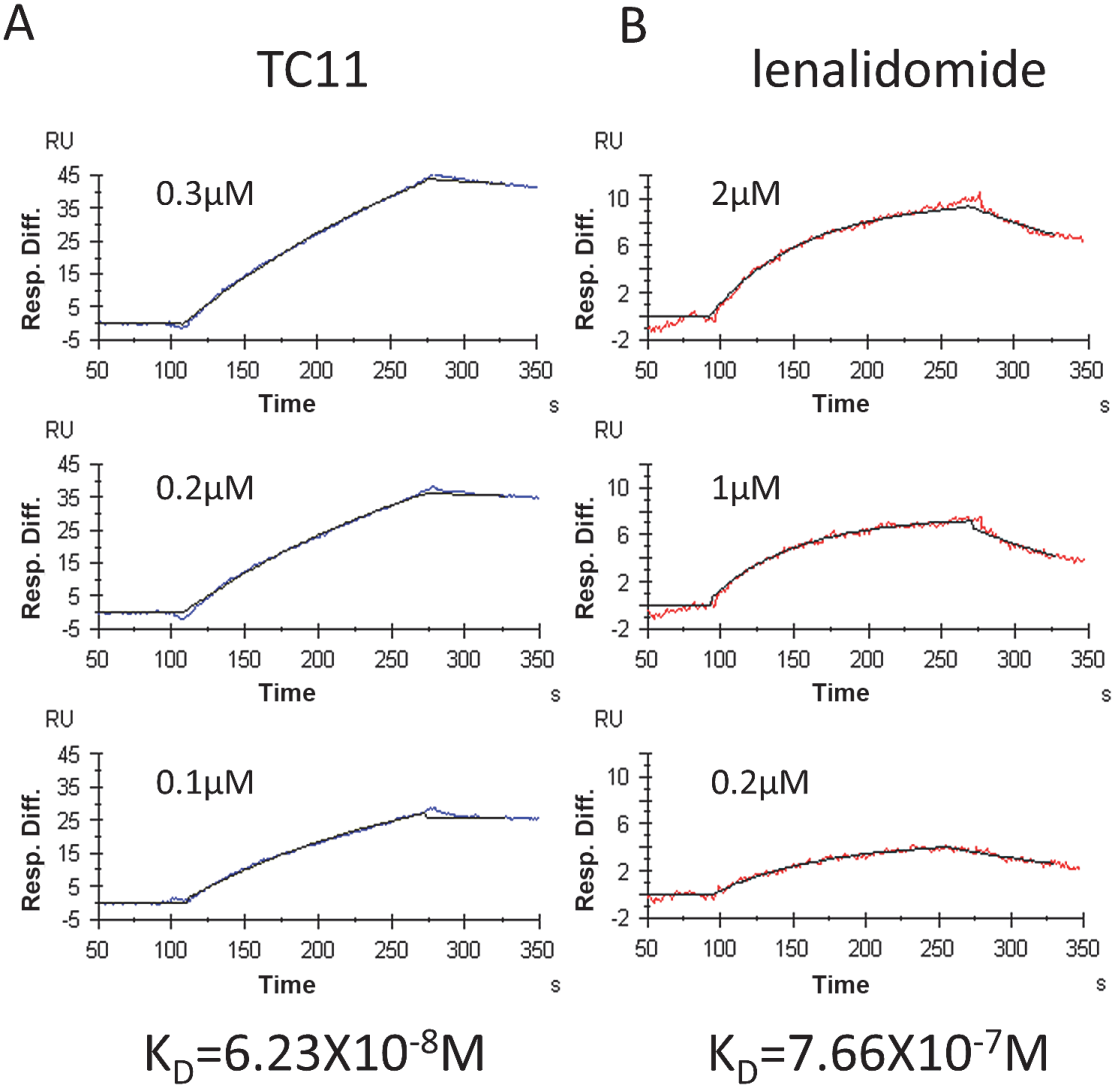
NPM1 interacts with TC11 and lenalidomide. Recombinant NPM1 was expressed in *E. coli* and purified. The NPM1 obtained was immobilized onto the sensor chip NTA followed by an SPR analysis. Panels A and B show representative biosensorgrams of TC11 and lenalidomide, respectively.

We previously identified NPM1 as one of the TC11-binding proteins, by mRNA display. In our previous observation, TC11 bound to the NH2-terminal 183-amino-acid (aa) region of monomeric NPM1. In the present study, we assessed the binding capacity of both TC11 and lenalidomide to the full length of NPM1. The SPR analysis showed that not only TC11 but also lenalidomide could bind to NPM1 (Fig. 5A,B). The K_D_ values of TC11 and lenalidomide were 6.23×10^−8^M and 7.66×10^−7^M, respectively.

## Discussion

MM remains difficult to treat and cure, and MM patients with high-risk cytogenetic changes in particular have shown very poor prognosis when treated with existing drugs. To overcome this unmet clinical need, in previous work we screened phthalimide derivatives and identified TC11 as a novel candidate drug for MM. TC11 is different from other IMiDs in chemical structure, lacking glutarimide moiety. That is, TC11 is a phthalimide derivative containing a benzene ring instead of a glutarimide structure (Fig. 1A). In the present study, we observed that TC11 inhibited the growth of MM cell lines, even though some of the myeloma cells harbored high-risk chromosomal abnormalities such as del17, or t(4;14). In addition, growth inhibition of bone marrow cells obtained from the relapsed MM patients was also examined. TC11 more or less inhibited proliferation of myeloma cells obtained from all the patients. Patient #3 showed t(4;14), and patient #2 had extramedullary disease in the liver. Even though these two patients were high-risk cases, TC11 regulated proliferation of the tumor cells (Fig. 1B). Moreover, the growth of total colony-forming cells was not suppressed by the treatment with 1–5 µM of TC11 even though TC11 significantly inhibited the growth of all MM cell lines in such concentrations, suggesting low hematological toxicity. It is speculated that myeloma cells may proliferate more dependently on TC11-associated molecules such as NPM1. Further study is needed to discriminate the molecular function of TC11 on myeloma cells from that on normal hematopoietic cells.

We also confirmed an *in vivo* anti-myeloma effect of TC11 by using intraperitoneal injections of TC11 to KMS34 and KMS11-bearing ICR/SCID mice. The tumor growth in these mice was significantly delayed by the administration of 20 mg/kg or higher dose of TC11. The histopathological examination revealed apoptosis of MM cells, which is consistent with our previous study showing that TC11 induces apoptosis through the activation of caspase 3, 8 and 9 *in vitro* [13]. In addition, no systemic side effects including weight loss occurred.

Our pharmacokinetics study indicated that TC11 was completely eliminated from the plasma at 4 h after the injection of a low dose (20 mg/kg) of TC11 and at 8 h after the injection of a high dose (100 mg/kg) of TC11. These data suggest that TC11 would not accumulate in patients by the administration of TC11 twice at a 3-day interval. To obtain stronger anti-tumor effects, it might be necessary to develop a new drug-delivery system or change the schedule of drug administration.

Lytic bone disease in MM is thought to be associated with both enhanced activity of osteoclasts and impaired function of osteoblasts. It was reported that bortezomib could cause an increase in bone formation by activating osteoblastic function, which leads to the improvement of osteolytic lesions [21]. Here, we found that TC11 inhibited both the maturation and function of osteoclasts. Osteoclasts are differentiated from hematopoietic stem cells through stimulation with M-CSF and RANKL. Under normal conditions, the balance between bone formation and bone resorption is well-controlled.

However, MM cells produce macrophage inflammatory protein-1 (MIP-1), which stimulates osteoclasts to express receptor activator of nuclear factor-κB (RANK). RANK interacts with RANKL on stromal cells or osteoblasts. This interaction leads to the activation of nuclear factor-kappa B (NF- κB) and nuclear factor of activated T cell c1 (NFATc1) via an activation of AP-1 [22]. In our M-CSF and RANKL-stimulated mouse bone marrow model, we observed that the differentiation of osteoclasts was significantly suppressed by at least 0.5 µM TC11. We also found that the function of osteoclasts was reduced by 3 µM TC11. These anti-osteoclastic effects might be characteristic of TC11, because these effects were not seen in the cells treated with thalidomide nor bortezomib. We also found that this effect was not due simply to the induction of apoptosis of the bone marrow cells, but rather occurred by a direct inhibition of osteoclast maturation, since apoptosis of osteoclasts was not observed after treatment with 3 µM TC11 (data not shown). It would be worthwhile to investigate the effects of TC11 on the expression of osteoclast-related genes such as AP-1.

To clarify the mechanism of TC11’s anti-myeloma effect, we investigated the effects of TC11 on NPM1, since we have found that NPM1 is one of the binding targets of TC11 by an mRNA display (i.e., the IVV method) [13]. In the present study, the results obtained by the SPR analysis indicated that TC11 binds to the full length of NPM1, with the *K*_D_ value of 6.23 × 10^−8^ M. However, the pharmacological significance of the binding of TC11 to NPM1 has not been elucidated.

NPM1 is a nuclear phosphoprotein which plays a variety of roles in cancer cells by affecting DNA repair, centrosome duplication, and molecular chaperoning [23–25]. NPM1 is also important in hematopoiesis by controlling the cell cycle of hematopoietic progenitor cells [26]. In addition, mutation of NPM1 is found in one-third of acute myelogenous leukemia (AML) patients, which changes the localization of NPM1 from the nucleus to the cytoplasm [27–29].

Little is known about the role of NPM1 in MM patients. To our knowledge, there is only one report on NPM and MM, showing an overexpression of NPM1 in hyperdiploid MM cells [30]. All of the MM cell lines we used in the present study expressed NPM1 proteins, but NPM1 gene mutation was not observed in these MM cell lines, and TC11 did not cause an accumulation of NPM1 in the cytoplasm of KMS34 as seen in leukemic cells (data not shown).

Another possibility explaining the role of NPM1 in TC11’s effects is that TC11 blocks the oligomerization of NPM1, since the binding site of TC11 contains the oligomerization domain of NPM1. NPM1 normally exists in oligomer form [31]. Therefore, TC11 might affect myeloma cells by blocking the oligomerization of NPM1 and inhibiting its various functions. Qi et al. showed that the inhibition of the oligomerization of NPM1 by a small-molecule inhibitor, NSC348884, induces the apoptosis of cancer cells through the activation of p53 [32]. Balusu et al. reported that NSC348884 could induce the apoptosis of AML cell lines [33].

It is also possible that TC11 impairs centrosomal disruption by binding to NPM1, because NPM1 has been reported to be indispensable to normal centrosomal duplication [34]. The NPM1 gene is located on chromosome 5q35, which is occasionally deleted in myelodysplastic syndrome (5q^−^MDS) [35]. Considering our observation that lenalidomide could also bind to NPM1, the contribution of these interactions to the anti-tumor effects should be further investigated.

We then analyzed the effects of TC11 on α-tubulin’s structure, since we identified α-tubulin as another TC11-binding protein using the IVV method in our previous work [13]. Alpha-tubulin is a component of microtubules that is important for cell division and cellular transport. Drugs that alter the formation of microtubules, such as vinca alkaloids, have been used in cancer therapy. However, the target sites and effects of these drugs on microtubules are different [36, 37]. In the case of TC11, we observed abnormal α-tubulin fragmentation in TC11-treated KMS34 cells (Fig. 4), which suggests that the apoptosis induced by TC11 might be triggered by an abnormal formation of microtubules. In the previous report, knockdown of NPM1 gene induced centrosomal disruption followed by activation of caspase-9 and significant reduction in cell viability [12]. Therefore, it is speculated that the principal actions of TC11 on myeloma cells are centrosomal disruption and abnormal microtubule assembly by binding to NPM1 and α-tubulin, respectively. Consequently, TC11-treated myeloma cells fall into mitotic catastrophe because NPM1 and tubulin families are key molecules for cell division. After these series of actions initiated by TC11-treatment, those cells caused apoptosis. As a next step to clarify exact signaling pathway after binding of TC11 to NPM1, we are trying to knock-down of NPM1 gene in myeloma cells and examine the effects of TC11 on oligomerization and phosphorylation of NPM1 in myeloma cells.

Cereblon (CRBN: cerebral protein with ion protease), a component of the E3 ubiquitin ligase complex, has been identified as a target of thalidomide-mediated teratogenicity [38, 39]. It was shown that not only thalidomide but also lenalidomide and pomalidomide bind to CRBN, attenuating the proliferation of myeloma cells through several pathways such as the down-regulation of autoubiquitination of CRBN [40–41]. However, our experiments using the IVV method failed to screen CRBN as a TC11-binding protein. The difference might be explained by the structure of TC11, because CRBN was reported to bind to a glutarimide moiety of lenalidomide or pomalidomide, which was not included in TC11. On the other hand, NPM1 may bind to the phthalimide moiety which is a common component of TC11 and other IMiDs, since our data showed both TC11 and lenalidomide could bind to NPM1.

Thus, TC11 exerts its anti-myeloma effect via molecular interactions which do not involve CRBN. In addition, TC11 does not form racemate and is expected to lack teratogenicity. The results of our present study suggest that new phthalimide derivatives other than thalidomide, lenalidomide and pomalidomide could be developed by drug designing for the treatment of MM.

In conclusion, we have demonstrated that TC11, a novel phthalimide derivative, has anti-tumor activity against MM cells with high-risk genetic abnormality including del 17p and t(4;14), *in vitro* and *in vivo.* This novel compound also down-regulates the differentiation and function of osteoclasts. Our data provide a strong preclinical rationale for TC11 as a safe and effective drug for the treatment of high-risk MM patients with bone disease. The actions of this drug relating to α-tubulin and NPM1 remain to be further investigated.
